# Roussel Uclaf Causality Assessment Method for Drug-Induced Liver Injury: Present and Future

**DOI:** 10.3389/fphar.2019.00853

**Published:** 2019-07-29

**Authors:** Gaby Danan, Rolf Teschke

**Affiliations:** ^1^Pharmacovigilance Consultancy, Paris, France; ^2^Department of Internal Medicine II, Division of Gastroenterology and Hepatology, Klinikum Hanau, Academic Teaching Hospital of the Medical Faculty, Goethe University, Frankfurt, Germany

**Keywords:** pharmacovigilance, DILI, drug-induced liver injury, RUCAM, Roussel Uclaf Causality Assessment Method, prospective studies

## Abstract

Among the causality assessment methods used for the diagnosis of drug-induced liver injury (DILI), Roussel Uclaf Causality Assessment Method (RUCAM) remains the most widely used not only for individual cases but also for prospective and retrospective studies worldwide. This first place is justified by the characteristics of the method such as precise definition and classification of the liver injury, which determines the right scale in the scoring system, precise definition of the seven criteria, and the validation approach based on cases with positive rechallenge. RUCAM is used not only for any types of drugs but also for herbal medicines causing herb-induced liver injury, (HILI) and dietary supplements. In 2016, the updated RUCAM provided further specifications of criteria and instructions to improve interobserver variability. Although this method was criticized for criteria such as the age and alcohol consumption, recent consensus meeting of experts has recognized their value and recommended their incorporation into any method. While early studies searching for DILI in large databases especially in electronic medical records were based on codes of diseases or natural language without causality assessment, the recommendation is now to include RUCAM in the search for DILI/HILI. There are still studies on DILI detection or the identification of biomarkers that take into consideration the cases assessed as “possible,” although it is well known that these cases reduce the strength of the association between the cases and the offending compound or the new biomarker to be validated. Attempts to build electronic RUCAM or automatized application of this method were successful despite some weaknesses to be corrected. In the future, more reflections are needed on an expert system to standardize the exclusion of alternative causes according to the clinical context. Education and training on RUCAM should be encouraged to improve the results of the studies and the day-to-day work in pharmacovigilance departments in companies or in regulatory agencies. It is also expected to improve RUCAM with biomarkers or other criteria provided that the validation process replaces expert opinion by robust standards such as those used for the original method.

## Introduction

The diagnosis of drug-induced liver injury (DILI) as any other disease needs to be supported by strong criteria. Until 1993, the main approach was the global introspection, also called expert opinion (EO), with unstructured arguments, absence of score, and no validation or general methods used in pharmacovigilance for any adverse drug reaction. A more objective method was needed and accepted by Council for International Organizations of Medical Sciences (CIOMS) as a topic for consensus meetings organized in 1990 and 1991 (references in Danan and Teschke, 2016). Later on, the Roussel Uclaf Causality Assessment Method (RUCAM) was created and validated with positive rechallenge cases (reference in Danan and Teschke, 2016), but some confusion remains on its name. This came from insufficient reading or misunderstanding of the original article. RUCAM was partly built on the results of consensus meetings, but many criteria were added and the scoring system was established and validated by the Roussel Uclaf team only. CIOMS did not want to endorse RUCAM because the method was not fully established by the members of the consensus meetings. RUCAM should therefore be named as such and not after CIOMS. However, even in a guideline on DILI (Andrade et al., 2019), the name of the method was different according to the chapters. Despite a slow start between 1993 and early 2000s, RUCAM became the most widely used method to support DILI diagnosis in different settings that prompted an updated version to further improve the results (Danan and Teschke, 2016). Several reasons concurred to this situation (Danan and Teschke, 2018): definition and classification of a liver injury, precise criteria, a scoring system, and the validation approach of the original method. RUCAM is now used not only for the diagnosis of DILI in individual cases, case series, registries, or epidemiological studies involving any types of drugs, herbal medicines, or dietary supplements but also as automated RUCAM and other settings such as the modern approach of searching for DILI in electronic medical records (EMRs).

The objectives of this article are twofold: first, to describe, comment on, and highlight how the current utilization of RUCAM can be improved and, second, to consider the future applications of RUCAM beyond individual cases to detect hepatotoxicity of any types of compounds administered to humans.

## Literature Search and Data Review

Published reports were systematically searched in electronic databases of Medline (source PubMed) from 2014 using the search terms: RUCAM, Roussel Causality Assessment Method, DILI, drug-induced liver injury, pharmacovigilance, and CIOMS. From each searched segment, the publications of the first 30 hits were analyzed and selected for reports in English language. Before the final analysis, the publications were assessed regarding the clinical quality and data completeness. The search was completed on 4 April 2019.

## Current RUCAM Use

### Why and Where RUCAM Is Used

RUCAM is fairly unique and should be seen in the context of other CAMs to be used in suspected DILI cases and described with their weaknesses (Teschke and Danan, 2018a). In short, to assess DILI, the least is to consider CAMs that are liver specific. Indeed, the general CAMs as proposed by WHO UMC or Naranjo (NAR) are designed to assess any adverse drug reactions, but specific timing, precise definition of dechallenge or rechallenge, list of alternative causes to exclude, and a scoring system are lacking. Furthermore, these methods were not validated or validated against an unstructured opinion. None of them was found better than liver-specific methods. Except the global introspection used by the Drug-Induced Liver Injury Network (DILIN) in the USA (Hayashi et al., 2015), the liver-specific CAMs directly derive from RUCAM, either with some changes to “simplify” RUCAM such as Maria and Victorino (MV) method or to add a controversial diagnostic laboratory test (Lymphoblastic transformation test) as a criterion in the DDW-J only used in Japan by few experts (Das et al., 2018). The strength of RUCAM comes from the precise definition of the criteria and the validation method based on cases with positive rechallenge. RUCAM has been conceived as a step-by-step method similar to a diagnostic approach. It was therefore important to define a liver injury to start off the process and to classify the liver injury because the time to onset and the time course of the biochemical markers are not identical for hepatocellular injury and a cholestatic/mixed liver injury. The causality assessment criteria and the time course of the usual biomarkers for hepatocellular and cholestatic/mixed liver injury are different as indicated in RUCAM. The details are given in the updated RUCAM (Danan and Teschke, 2016) as well as in the work instructions (electronic supplementary material in Danan and Teschke, 2018). Efforts in Europe brought the fascinating topic of DILI back to the roots by consolidating DILI-related science (Björnsson, 2014; Björnsson, 2016; Danan and Teschke, 2016; Andrade et al., 2016; Danan and Teschke, 2018), where DILI was early considered as a challenging disease requiring robust causality assessment method such as RUCAM. This method was used in many prospective DILI studies in Europe and contributed to evidence-based characterization of DILI features. Much support for deep analysis of DILI cases and causality assessment by the updated RUCAM was provided by recognized experts in DILI (Sarges et al., 2016; Shahbaz et al., 2018; Real et al., 2019). Despite strong recommendations, reviews and reports on DILI characteristics are still published each year based on a simple opinion of the author(s) and accepted by reviewers leading to the impression that DILI is not seriously taken. In 2016, a list of DILI cases with validated DILI diagnoses was published, providing a large number of cases published by authors worldwide (Danan and Teschke, 2016). Additional reports were published in many countries, including the USA (Cheetham et al., 2014) and China where RUCAM is included in the national guidelines on DILI (Yu et al., 2017; Shen et al., 2019). Until early 2019, the total number of RUCAM-based DILI cases reported since 1993 is close to 50,000 cases ranking RUCAM as the most commonly used CAM in DILI (Teschke, 2019, in press). Beyond chemical drugs RUCAM has been used for other compounds such as herbal medicines or dietary supplements (Teschke and Eickhoff, 2015; Teschke et al., 2016; Zhu et al., 2016).

### RUCAM in Clinical Settings

In clinics, for general practitioners or specialists confronted with ALT elevation with or without jaundice but without obvious cause, one of the most frequent issue is to diagnose a DILI. As shown in the UK between 1998 and 2014, the most frequent cause of jaundice and ALT above 400 IU/l on 1,000 consecutive patients in clinics in elderly patients is DILI (Vine and Dalton, 2015). Here, RUCAM is helpful to follow a step-by-step approach, collect prospectively the relevant data, score each suspected drug/herbal medicine, and finally come up with a diagnosis based on transparent criteria and score that allow for a reassessment by colleagues. This approach is supported by independent teams in many countries (Kullak-Ublick et al., 2017). For each patient where DILI is suspected, a RUCAM work sheet should be systematically completed and be part of the patient file.

Clinical trials should not be excluded from the RUCAM use as it was wrongly suggested (Regev et al., 2014). There is no argument to reject RUCAM in clinical trials but just the opposite. Indeed, in this context, it is usual to collect specific and relevant data, and normally, the suspected DILI cases are reported on ongoing basis provided that the flow chart for the management of ALT elevation is available for the investigators and the monitors. It is argued that RUCAM is not adapted to clinical trials although the criteria for causality assessment do not differ qualitatively and quantitatively from the postmarketing setting. The data collected for each case are best used with RUCAM to achieve a correct diagnosis of DILI as shown in a study using a RUCAM-based automated method applied to a dataset from clinical trials (Scalfaro et al., 2017). Moreover, the RUCAM criteria can serve to reach consensus in case of difficult and critical situation in drug development (Teschke and Danan, 2016).

RUCAM has been criticized for the tendency to lower the final score in case of death or liver transplantation due to acute liver failure since the dechallenge is not assessable. This is also true for any CAMs, including EO, but with RUCAM, the exclusion of the alternative causes, if done properly, will increase the final score of the suspect drug. In the case of multiple drugs, RUCAM allows for ranking the suspect drugs. If the drug is indispensable and after a consensus of experts, there is sometimes no other solution than to perform a drug rechallenge test and assess the results according to the strict criteria as provided in RUCAM (Danan and Teschke, 2016; Danan and Teschke, 2018).

### Automated RUCAM

As an algorithm with a scoring system, it was tempting to automatize RUCAM. The incorporation of the updated RUCAM in an electronic program would accelerate the evaluation process of large case numbers and likely reduces interrater variability. At the same time, transparency of the evaluation with RUCAM is further enhanced. This was performed by an expert team in DILI to make the causality assessment independent from individuals (Cheetham et al., 2014). This attempt was a success, with a high agreement between the automatized RUCAM and manual RUCAM scoring. However, the exclusion of alternative causes is recognized as a difficult criterion that should take into consideration the clinical context (Teschke and Danan, 2018b). In the future, a sort of expert system should be built to support this criterion. In addition, the authors rightly suggested to include automated RUCAM into EMRs for the detection of DILI in hospital-based population.

To facilitate case evaluation of suspected DILI in routine pharmacovigilance, an algorithm was proposed based on RUCAM with many supportive tables and named Pharmacovigilance-Roussel Uclaf Causality Assessment Method (PV-RUCAM) (Scalfaro et al., 2017). This project was prompted because, in spontaneous reports, data are usually incomplete and PV professionals are not necessarily experts in DILI. The performance of this method was compared in different settings with regard to its applicability and differentiation capacity. The score was applied in two datasets of individual case safety reports (ICSRs) extracted randomly from clinical trial reports and a third dataset of electronic health records from a global PV database. The results of PV-RUCAM were compared to the original RUCAM and EO and showed 100% sensitivity, 91% specificity, 25% positive predictive value, and 100% negative predictive value. Similar to the original reproducibility test of RUCAM, there was high inter-rater agreement (Kw = 0.79) between the two PV-RUCAM assessors. The authors concluded that, compared to other methods, PV-RUCAM is of great help in incomplete ICSRs assessed for DILI by nonexpert PV professionals. This attempt to build a RUCAM-based automated algorithm to be used in any pharmacovigilance department is encouraging and should trigger further research on RUCAM: first, prospective validation of this algorithm as proposed by the authors and, second, fully automatized RUCAM with a sophisticated expert system behind the algorithm to consider (almost) all the clinical situations.

Computerization of causality assessment method was also recently addressed (Tillmann et al., 2018) to decrease variability between raters and improve the results of a CAM. Additional criteria beyond those already included in RUCAM were proposed such as genetics, race, gender, or drug signatures, but the current knowledge on these factors is not strong enough in terms of sensitivity and specificity to be included and scored in any CAM. More prospective studies are needed to add new and validated criteria.

The use of automated RUCAM should be encouraged in prospective and retrospective studies especially in large databases with thousands of potential DILI cases, as they exist in the form of EMR in hospitals (Hunt, 2018) or national health insurance registries. However, it should be used concurrently with strict DILI definitions and verification of the diagnosis in a sample of positive and negative cases.

### RUCAM in Databases, DILI Registries, and Epidemiological Studies

A study in the USA at Michigan University developed a novel text searching tool to identify DILI cases in EMR (Heidemann et al., 2017). RUCAM and EO were applied to suspected DILI cases with good agreement between these methods despite the fact that, in very few cases, the authors were unable to use RUCAM because of missing data on the time to onset of the liver injury. This so-called limitation of RUCAM is not substantiated because it is always possible to assign the lowest score to this criterion in absence of information (Danan and Teschke, 2016). Furthermore, missing data are also an issue for the global introspection method, where it constitutes a limiting step unless assumptions are made that could also be used in RUCAM. A study in Japan using RUCAM and a Japanese method DDW-J to build an algorithm for DILI detection in a medical information database showed an expected agreement between the methods since DDW-J derives from RUCAM and called for further studies on DILI in large EMR database (Hanatani et al., 2014). A meta-analysis of algorithms to identify DILI in EMR (Tan et al., 2018) in 29 studies between 1993 and 2016 included causality assessment methods: EO in 16, RUCAM in 8 (starting in 2000), WHO in 1, RUCAM and WHO in 1, DDW-J in 1 and none in 2 studies. The positive predictive value of DILI detection algorithms calculated on 25 studies was low, ranging from 1.0 to 40.2%. These results were due to considerable variability in case definition of DILI, causality assessment methods, diagnostic codes, and study drugs. Interestingly, the authors concluded that DILI detection algorithms could be improved by the adoption of the internationally agreed DILI definition, the use of the RUCAM, the screening of high-risk drugs, and use of natural language processing and machine learning algorithms. This conclusion was supported by a thoughtful editorial accompanying this article suggesting clues to improve DILI detection algorithms such as drug–drug interactions, drug combinations, patient factors, herbals and dietary supplements that would account for 15% of DILI cases, and the environment (Hunt, 2018).

The first DILI registries were based on EO or general methods used in pharmacovigilance, but very early DILI definition and validated liver-specific CAM were needed to include cases. The adoption of internationally agreed definition of biochemical thresholds in ALT, AST, ALP, and bilirubin and liver-specific CAM improved considerably the reliability of the registries and any type of epidemiological studies. CIOMS then RUCAM introduced liver test thresholds and liver injury classification before causality assessment criteria. RUCAM with its algorithm and scoring system provide easy tools for epidemiologists to ensure homogeneity and reliability of the results. Despite this advance in searching for tools in DILI, the LiverTox database has not systematically used RUCAM but preferably EO to analyze the published cases. In a study based on the original and the updated RUCAM, the quality of a sample of the LiverTox DILI cases was assessed. As a result, some cases included in this database are likely not DILI due to insufficient data quality (Björnsson, 2016; Björnsson and Hoofnagle, 2016). Indeed, this issue was also discussed by others (Real et al., 2019; Teschke and Danan, 2018b) asking for improvements to continue to rely on LiverTox as a worldwide known and popular database on DILI characteristics.

RUCAM is widely used in epidemiological studies for the calculation of DILI incidence rates and to rank drugs according to their hepatotoxicity. Interestingly, a study to estimate DILI incidence in EMR database jumped over CAMs to use only DILI definition and temporally related criteria (Shin et al., 2013). As the selected codes excluded alternative causes, it was thought that DILI cases were correctly assessed for causality. The diagnosis was based on the assumption that the codes were valid. This was not verified on a sample of cases and constitutes one of the weaknesses of this study. It would have been preferable to use RUCAM as strongly suggested to ascertain the diagnosis of DILI and would make the incidence rates, DILI characteristics, and other calculations more reliable (Hunt, 2018). Another example in a large hospital database is a case–control study designed to detect hepatotoxicity of new drugs and quantify the risk of DILI, RUCAM was used to search for cases and assess causality (Douros et al., 2014). Other RUCAM-based incidence studies performed before 2014 in various countries are summarized in a recent review (García-Cortés et al., 2018). Many epidemiological studies were based on RUCAM to identify DILI cases among which it is worth quoting: registries in Spain, Iceland, Latin America (Bessone et al., 2019), or in hospital data bases in Korea in 2012, Mainland China (Shen et al., 2019), Japan (Aiso et al., 2019), Thailand (Sobhonslidsuk et al., 2016), in a DILI cohort study for liver transplantation (Baekdal et al., 2017), and in a prospective cohort study in India (Rathi et al., 2017). The latter study is a good example that, when RUCAM is used prospectively, the identification of DILI cases as well as causality assessment is easier and more rigorous, and the results case by case are transparent (Teschke and Danan, 2017). Likewise, a prospective cohort study in a tertiary center for liver disease based on RUCAM and compared to studies in other countries (Licata et al., 2017) showed that drug classes involved in DILI are similar in many countries. Wider dissemination of the RUCAM is supported by authors after a study in Brazil, realizing that some epidemiological studies are still based on DILI cases on simple opinion without definition of criteria (Becker et al., 2019).

### RUCAM for the Validation of Biomarkers and Risk Factors

To improve our knowledge on DILI, to detect hepatotoxicity of the new therapies such as immunotherapy, and to validate biomarkers and risk factors, high confidence in DILI cases is needed. It is therefore of the utmost importance to define DILI and use validated CAM such as RUCAM in studies testing new markers. Since RUCAM provides for each case the detail of the criteria and how the score has been reached, another expert will be able to reassess the cases. Although it has been repeatedly said that only “probable” (score 6–8) and “highly probable” (score >8) cases should be taken into consideration (Teschke et al., 2017), there are still studies incorporating “possible” cases into DILI cases (Russmann et al., 2014). It is frequently unknown whether these cases are “possible” due to insufficient data on alternative causes, as in retrospective studies, or to concomitant medications because they were given exactly at the same time as for the studied drug. Sometimes to increase the sensitivity of the test and sometimes by misunderstanding of the CAM, these “possible” cases are added to the positive cases. As a result, the reliability of the association is reduced between the DILI cases and the suspected offending compounds whether it is a drug, an herbal medicine, or a dietary supplement, and the conclusion will not be as convincing as it should be. Genetic and serum biomarkers studies are examples where DILI cases should not suffer approximation in the diagnosis (Tao et al., 2018). The link is commonly weak with studied markers, and it is tempting to add “possible” cases to reach the significant threshold (Liu et al., 2018). This approach should always be refused by reviewers and editors who are confronted with borderline results. Same applies to published DILI cases on a new drug or case series on established drugs where the authors based the assessment on their own opinion and not on RUCAM. The reviewers, as experts, should pay attention to these cases that could enter in DILI databases only because they are published and not more rigorously on the basis of objective criteria and transparent method. Moreover, if the publication includes few cases, the detailed score of RUCAM has to be included in the article or in a supplementary material to ensure the transparency of the assessment. Despite these recommendations (Danan and Teschke, 2018), a number of articles are still published only on the basis of the author’s opinion preventing a reassessment by the reviewer and the editor.

Similarly, regulators should base their evaluation and decision on validated DILI cases assessed with RUCAM. Few national agencies require such assessment when drug hepatotoxicity is discussed after the first case(s) of acute liver injury in postmarketing or clinical trials in drug development. Usually, this risk is considered high enough, depending on the severity and the incidence of the cases, to induce significant changes in the risk benefit balance that could result in a better use of the drug to reduce the risk of acute liver injury or a drug withdrawal from the market. Hepatologists and, more precisely, DILI experts should include an assessment with RUCAM in the drug evaluation and if not done to justify their position. This would ensure transparency of the drug evaluation and the regulatory decision.

## Future Use of RUCAM

### Education

The most important step for using RUCAM is to be trained with the tool. The method is simple and user-friendly. The user should follow a stepwise approach, and it is assumed that each criterion is understood according to straightforward instructions given in the supplementary material of the following article (Danan and Teschke, 2018). One of the properties of RUCAM is flexibility to accommodate almost all clinical situations even though the minimum information is missing. Some case studies need to be assessed with a senior user to understand the concept. Nothing new for a hepatologist, but for the staff working in a PV department, the basic education in hepatology is needed. Indeed, understanding the liver tests, the definition and the types of liver injury, the main causes of an acute liver injury, and the need to search for alternative causes including herbal and dietary supplements are necessary to apply intelligently RUCAM. It is worth training one or two staff members in a team to use RUCAM as the researchers do when a case series is to be identified in databases or to monitor a registry. The recent efforts to automatize RUCAM will be of considerable help.

### RUCAM-Based DILI Cohorts for International Harmonization

Although RUCAM is occasionally used retrospectively on DILI cases, it is recommended to use it prospectively, as it constitutes a guide to collect relevant data to assess causality. The example of a prospective study in India shows high quality of the data and subsequent reliability of the results (Rathi et al., 2017). On the opposite, in retrospective studies, the authors struggle to obtain data for causality assessment. This is true not only for RUCAM but also for other CAMs. The particularity of RUCAM is to be available in a worksheet with all the criteria needed to calculate the final score of the suspected DILI case. It is therefore easier to ask on ongoing basis the investigator/reporter for specific data and complete the score. With RUCAM, retrospective or prospective studies involving DILI will benefit for a harmonized approach. The language for international DILI studies could be provided by RUCAM. It would be of great scientific interest to gather the cases from databases in different countries. This cooperation would increase the number of cases and controls so that the power of the detection would increase in parallel as well as the identification of risk factors such as obesity, nonalcoholic fatty liver, diabetes mellitus, HLA variants, and genetic and serum biomarkers. Together with the preceding rule on the incorporation of “probable” and “highly probable” cases only would make significant progress in our knowledge on DILI.

### RUCAM Improvements

Responses to comments on the original version of RUCAM of 1993 (Hassan and Fontana, 2019) were provided and already implemented in the updated version (Danan and Teschke, 2016). Other comments were addressed in a summary table (Danan and Teschke, 2018) and in detailed work instructions (Electronic supplementary material in Danan and Teschke, 2018). The introduction of new biomarkers and inclusion into RUCAM were specifically discussed (Teschke et al., 2017) to focus on the method for the validation of new criteria. Another approach proposed by a DILI expert would be to combine RUCAM results with the DILI signature, i.e., the clinical and biochemical characteristics, of a specific drug as provided in articles or in specialized database such as LiverTox (Watkins, 2015). The weakness of this approach is to make sure that the DILI signature is correct and validated. Ongoing international initiatives are working on the modification or addition of criteria to RUCAM. The issue here is, first, the validation of the criteria and, second, the validation of the whole method after the changes. Instead of a qualitative approach of potential changes, it would be necessary to validate the modifications using a gold standard such as the one used to validate the original method with positive rechallenge cases. Although this validation method could be discussed, it has the value to be objective and reproducible and not to be based on EO, which is subjective by definition.

## Conclusion

**Figure 1:**
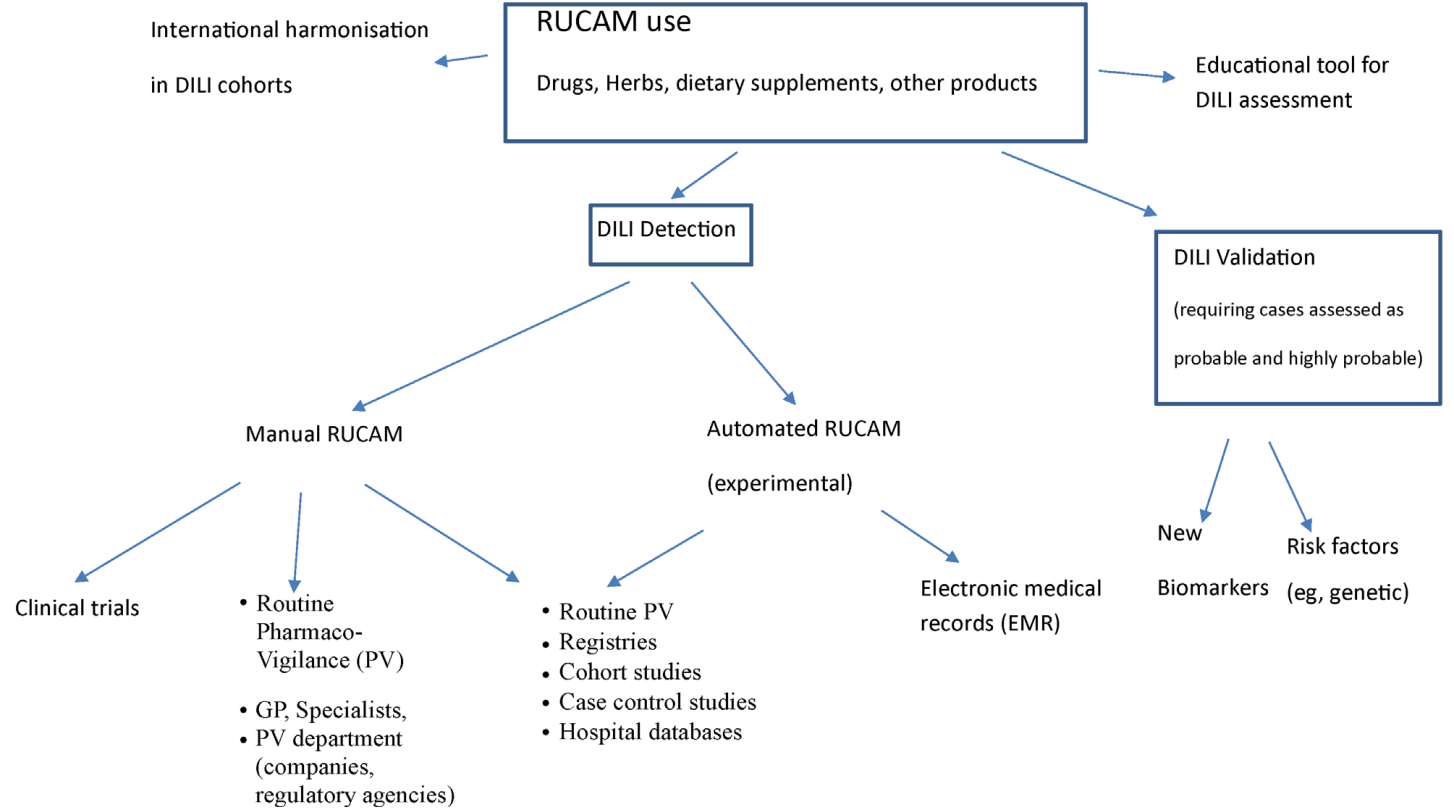
Current RUCAM use.

The analysis of the current RUCAM use shows that the method is adapted to the clinical situations as well as to prospective and retrospective studies involving DILI or searching for DILI (**Figure 1**). The method is also used for herb-induced liver injury (HILI) and intoxications to follow a rigorous guide and exclude the alternative causes. RUCAM is a reliable tool for the harmonization of international studies and regulators to help make a decision based on objective criteria in case of drug hepatotoxicity. It was also shown that RUCAM is adapted to liver injury induced by herbs or dietary supplements, which account for an increasing proportion of liver injuries especially in Asian countries. In the future, more educational efforts should be made to introduce the method in training programs for PV experts in companies or national agencies. More studies on automated RUCAM are needed to facilitate the RUCAM use by nonexpert PV professionals and to build expert system to search for alternative causes according to the clinical circumstances. Studies on DILI cases involving new therapies or biomarkers should take into consideration only the “probable” and “highly probable” cases assessed by RUCAM to be published or strengthen the association between DILI cases and the suspect drug/herb or the biomarker. More studies are warranted to search for RUCAM improvements by adding or modifying criteria, provided that validation process be based on robust standard, such as cases with positive rechallenge, as used for the original method. It is hoped that the use of RUCAM will further increase confidence on the studies performed for DILI detection with new drugs, herbs, and dietary supplements and the identification of risk factors and new biomarkers to be able to take the appropriate measures to reduce the risk of hepatotoxicity.

## Author Contributions

The authors equally contributed to the manuscript.

## Conflict of Interest Statement

The authors declare that the research was conducted in the absence of any commercial or financial relationships that could be construed as a potential conflict of interest.

The reviewer JBS declared a shared affiliation, though no other collaboration, with one of the authors, RT, to the handling Editor.
